# Temporal Dynamics of the Transcriptional Response to Dengue Virus Infection in Nicaraguan Children

**DOI:** 10.1371/journal.pntd.0001966

**Published:** 2012-12-20

**Authors:** Stephen J. Popper, Aubree Gordon, Minghsun Liu, Angel Balmaseda, Eva Harris, David A. Relman

**Affiliations:** 1 Department of Microbiology and Immunology, Stanford University, Stanford, California, United States of America; 2 Division of Epidemiology, School of Public Health, University of California, Berkeley, California, United States of America; 3 Division of Infectious Diseases and Vaccinology, School of Public Health, University of California, Berkeley, California, United States of America; 4 Department of Microbiology, Immunology, and Molecular Genetics, University of California Los Angeles and Cedars-Sinai Medical Center, Los Angeles, California, United States of America; 5 Laboratorio Nacional de Virología, Centro Nacional de Diagnóstico y Referencia, Ministry of Health, Managua, Nicaragua; 6 Department of Medicine, Stanford University, Stanford, California, and Veterans Affairs Palo Alto Health Care System, Palo Alto, California, United States of America; Duke University-National University of Singapore, Singapore

## Abstract

Dengue is the most prevalent mosquito-borne human illness worldwide. The ability to predict disease severity during the earliest days of the illness is a long-sought, but unachieved goal.

We examined human genome-wide transcript abundance patterns in daily peripheral blood mononuclear cell (PBMC) samples from 41 children hospitalized with dengue virus (DENV) infection in Nicaragua, as well as 8 healthy control subjects. Nine patients had primary dengue fever (DF1), 11 had dengue fever with serologic evidence of prior DENV infection, i.e., secondary dengue fever (DF2), 12 had dengue hemorrhagic fever (DHF), and 9 had dengue shock syndrome (DSS). We identified 2,092 genes for which transcript abundance differed significantly between patients on days 3–6 of fever and healthy subjects (FDR<1%). Prior DENV infection explained the greatest amount of variation in gene expression among patients. The number of differentially expressed genes was greatest on fever day 3 in patients with DF1, while the number in patients with DF2 or DHF/DSS was greatest on day 5. Genes associated with the mitotic cell cycle and B cell differentiation were expressed at higher levels, and genes associated with signal transduction and cell adhesion were expressed at lower levels, in patients versus healthy controls. On fever day 3, a set of interferon-stimulated gene transcripts was less abundant in patients who subsequently developed DSS than in other patient groups (p<0.05, ranksum). Patients who later developed DSS also had higher levels of transcripts on day 3 associated with mitochondrial function (p<0.01, ranksum). These day 3 transcript abundance findings were not evident on subsequent fever days.

In conclusion, we identified differences in the timing and magnitude of human gene transcript abundance changes in DENV patients that were associated with serologic evidence of prior infection and with disease severity. Some of these differential features may predict the outcome of DENV infection.

## Author Summary

Infection with dengue virus (DENV) causes dengue fever, the most prevalent mosquito-borne illness of humans worldwide. Tens of millions of cases occur annually; up to 500,000 patients develop additional life-threatening complications, including hemorrhage and shock. The clinical course of the disease evolves rapidly, making it difficult to identify patients at risk for severe disease and suggesting that biological events associated with the development of severe disease may be short-lived. We examined gene expression patterns in the blood of children hospitalized with DENV infection, and found that patients with differences in disease severity and history of previous DENV infection shared a common set of gene expression features, but the timing and magnitude of these features differed. In our study, prior DENV infection explained the greatest amount of variation in gene expression among patients. We discovered features of gene expression on day 3 that were associated with subsequent disease severity—features that were not apparent on subsequent days, emphasizing the importance of looking at temporal patterns of gene expression in acute infection.

## Introduction

As many as 100 million people are infected by dengue virus (DENV) each year, causing up to 40 million cases of dengue fever (DF). Approximately 500,000 individuals are hospitalized annually with DF and additional signs of hemorrhage, plasma leakage, low platelet count, and/or hypovolemic shock [1]. Identification and management of patients at risk for shock is difficult because the initial febrile phase of illness is similar in all patients, regardless of subsequent clinical severity, and because the onset of plasma leakage can lead to hypovolemic shock in less than 24 hours [2]. Understanding why there are differences in clinical severity and identifying early features associated with development of severe disease are therefore the subject of much-needed research.

Although severe disease can occur following an individual's first DENV infection (primary DENV infection), the single, most well-established epidemiologic risk factor for severe disease is a subsequent infection with a heterologous DENV serotype (secondary DENV infection). The severe disease in cases of secondary infection is thought to be due to enhanced entry of virus into Fcg receptor-bearing myeloid cells, mediated by non-neutralizing or sub-neutralizing anti-DENV antibodies, and/or aberrant activation of cross-reactive T-cells. Soluble factors and cell surface markers have also been associated with severe disease, but in general, the cellular and molecular events that occur after the initial virus-host interactions and contribute to clinical outcome are not well understood (reviewed in [3]).

Transcriptional profiling of blood cells has been used to characterize the acute host response to infection with a variety of pathogens. Host gene expression patterns have been linked to microbiological diagnosis, treatment response, and disease severity in a number of studies of systemic infectious and inflammatory disorders [4], [5], [6], [7], [8], [9], [10]. In particular, host gene expression patterns have been examined in groups of DENV-infected patients with differences in disease severity in an attempt to identify markers and pathways associated with the development of severe disease [8], [11], [12], [13], [14], [15], [16]. Despite common themes, findings from these studies reveal important differences in patterns of gene expression; for example, some but not all studies have reported higher transcript abundances for interferon-stimulated genes in patients with less severe disease.

Given the dynamic nature of dengue and other acute infectious diseases, predictive features based on gene expression may be transient and may be missed in cross-sectional studies that group together patients seen on different days of illness or that examine expression patterns on only one day of the illness. In the case of dengue, differences in the response to primary and secondary infections may complicate efforts to identify signatures associated with severe disease. Previous studies have either involved patients who had all experienced a prior DENV infection or have not distinguished between patients with primary and secondary DENV infection. In Nicaragua, a substantial proportion of pediatric patients hospitalized with dengue have not experienced a prior DENV infection [17]. In addition, one serotype tends to dominate each epidemic season [18]. This situation affords a comparison of the host responses to a given viral serotype in patients with primary and secondary DF (DF1 and DF2), and in patients with differences in clinical severity. The goals of this study were to characterize the temporal evolution of the host transcriptional response to DENV infection, compare the responses in patients with primary and secondary infections, and identify features associated with subsequent clinical severity.

## Methods

### Ethics statement

All work with human subjects was approved by the Institutional Review Boards of the University of California, Berkeley, and the Nicaraguan Ministry of Health, and by the Stanford University Administrative Panel on Human Subjects in Medical Research. Parents or legal guardians of all subjects provided written informed consent, and subjects 6 years of age and older provided assent.

### Study population and sample collection

Blood samples were collected from pediatric patients with DENV infection at the Hospital Infantil Manuel de Jesús Rivera (HIMJR) in Managua, Nicaragua between September 2005 and March 2007. Both DENV1 and DENV2 were circulating in Managua during the 2005–2006 dengue season; DENV2 was the predominant serotype in 2006–2007 [18], [19]. Patients were recruited into the study upon presentation to the hospital. Enrollment criteria consisted of hospitalization, age less than 15 years, and presentation with an acute febrile illness of less than 7 days duration with one or more of the following symptoms or signs at the time of evaluation: headache, arthralgia, myalgia, retro-orbital pain, positive tourniquet test, petechiae, and signs of bleeding. Serological, virological, and molecular biological assays to detect DENV infection were performed at the National Virology Laboratory of the Ministry of Health in Managua. A positive case was defined by detection of viral RNA by RT-PCR directed to the capsid gene [20], [21], virus isolation in C6/36 cells [22], IgM seroconversion between acute and convalescent-phase samples, and/or a 4-fold or greater increase in total anti-DENV antibodies between acute and convalescent-phase samples as measured by inhibition ELISA [20], [23]. Primary DENV infection was defined by an antibody titer by inhibition ELISA [20], [23] of <20 in acute samples or <2,560 in convalescent samples. Secondary DENV infection was defined by an antibody titer by inhibition ELISA of ≥20 in acute samples or ≥2,560 in convalescent samples [20], [24]. This technique was previously validated for classification of primary and secondary DENV infections as compared to the hemagglutination inhibition assay [22].

A standardized questionnaire was administered to collect demographic and clinical information at the time of admission, and clinical data during hospitalization were prospectively collected using standardized forms that were verified via chart review. The first day of fever (fever day 0) was determined by parental or guardian report. Venous blood was drawn for clinical tests and serological, virological and molecular biological dengue diagnostic assays when patients presented to the Infectious Diseases Unit, daily during hospitalization, at the time of discharge, and when possible, approximately two weeks after symptom onset. Six ml of blood were collected in CPT tubes (Becton-Dickenson, Franklin Lakes, NJ); the transport temperature (∼28°C) and times of sample collection, transport, reception, and processing were strictly controlled using personal data assistants (PDAs) with barcode scanners. Peripheral blood mononuclear cells (PBMCs) were isolated from whole blood within four hours of collection; half of each sample (average of 7×10^6^ cells) was mixed with Trizol and stored at −80°C for RNA analysis. Disease severity was classified according to the 1997 World Health Organization classification scheme as having DF, Dengue Hemorrhagic Fever (DHF), or Dengue Shock Syndrome (DSS) [25]. Blood samples from healthy subjects were collected as part of a separate prospective cohort study in which healthy children in the general population were enrolled without regard to dengue status and, as part of the study, subjected to annual blood sample collection [26]. At the time of collection of these blood samples, children did not have fever or other signs or symptoms of illness. PBMCs were prepared as above.

### DNA microarray sample processing

500 ng of total RNA from each sample and a standard reference RNA (Universal Human Reference RNA, Stratagene, La Jolla, CA) were amplified using the MessageAmp II-96 amino-allyl amplification kit (Ambion Cat# 1753). Transcripts in the samples and in the reference were then labeled with fluorescent dyes (Cy5 and Cy3, respectively, GE HealthSciences Cat# RPN5661), mixed together, and hybridized to HEEBO oligonucleotide microarrays printed on poly-L-lysine -coated glass slides. The HEEBO microarrays contained ∼44,000 70-mer oligonucleotide probes, representing ∼30,000 unique genes. A detailed description of this probe set can be found at (http://microarray.org/sfgf/heebo.do). Dye-labeled RNA was fragmented (Ambion Cat# 8740) and then diluted into 50 µl of solution containing 3× SSC, 25 mM Hepes-NaOH (pH 7.0), 20 µg of human Cot-1 DNA (Invitrogen Cat# 15279011), 20 µg of poly(A) RNA (Sigma Cat# P9403), 25 µg of yeast tRNA (Invitrogen Cat# 15401029), and 0.3% SDS. The sample was incubated at 70°C for 5 min, centrifuged at 14,000 rpm for 10 min in a microcentrifuge, then hybridized to the microarrays at 65°C using the HYBEX hybridization system (SciGene) for 14 hours. Following hybridization, microarrays were washed in a series of four solutions containing 2× SSC with 0.05% SDS, 2× SSC, 1× SSC, and 0.2× SSC, respectively. The first wash was performed for 5 min at 60°C; subsequent washes were performed at room temperature for 2 min each. Following the last wash, the microarrays were dried by centrifugation. Washes and dryings were carried out in a low-ozone environment (Hybex with ozone scrubber) to prevent destruction of Cy5 dye.

### Scanning and data processing

Microarrays were interrogated using the AxonScanner 4200AL (Molecular Devices). Each element was located and the associated data recorded using SpotReader (Niles Scientific). The microarray data were submitted to the Stanford Microarray Database for further analysis [27]. Background measurements were calculated using the Edwards correction, and data were then normalized using a 2D spatial loess estimation [28]. Data were filtered to exclude elements that did not meet the following criteria: a regression correlation of ≥0.6 between Cy5 and Cy3 signal over the pixels comprising the array element, an intensity/background ratio ≥2 in at least one channel, and <90 percent of pixels saturated in both channels. The microarray data are available from Gene Expression Omnibus (GEO accession number GSE38246) (http://www.ncbi.nlm.nih.gov/geo/).

### Data analysis

Principal components were identified using singular value decomposition [29]. The projection of a transcript on a principal component was considered significant for enrichment analyses if its value was at least one standard deviation from the mean projection value of all transcripts in the dataset. The Database for Annotation, Visualization, and Integrated Discovery (DAVID) was used to identify Gene Ontology (GO) terms, biological pathways from the Kyoto Encyclopedia of Genes and Genomes (KEGG), and Swiss-Prot keywords associated with co-expressed gene sets identified in our analysis [30]. To minimize the prevalence of redundant GO terms, the functional clustering tool in DAVID was used to group GO terms with overlapping membership, using the “medium” setting; the most significant GO term in each group was then chosen to represent each functional cluster. GO terms and Swiss-Prot keywords were considered significant if the p-value was <0.05 after Benjamini correction for multiple testing. NextBio software and database (http://nextbio.com) were used to identify cell types and other array datasets associated with genes in our analysis and to establish corresponding p-values [31]. Significance Analysis of Microarrays (SAM) was used to identify genes associated with differences between patient groups, or between patient groups and healthy controls [32]. Correlations between relative gene expression levels and clinical parameters were calculated using perl-based scripts; the significance of Pearson correlation coefficients was calculated after deriving a null distribution through permutation of sample labels [5]. Potential differences in clinical parameters and median expression of specific gene sets were tested using the Wilcoxon rank sum test and STATA v.10 (STATA, College Station, TX).

## Results

### Serological history of exposure to dengue virus explains the greatest amount of variation in patterns of gene expression

A review of the clinical data associated with subjects in this study indicated that patients who developed dengue hemorrhagic fever (DHF) or dengue shock syndrome (DSS) after hospitalization were more likely overall to have been hospitalized on fever day 4 than on any other day of illness. We therefore chose the PBMC samples collected on day 4 from DHF/DSS cases for our initial analyses and identified patients with dengue fever (DF) matched for sex, age, and days of fever. To explore how transcriptional responses varied from day to day, we included samples obtained on other days of fever from these patients, including multiple samples from some patients (median of 2 samples per patient, range 1–5 samples). We also included samples from both patients with primary and secondary DF (DF1 and DF2), as defined by results of serological tests (see Table 1 and Fig. S1). After sample processing, array hybridization, and quality control of the data (see Materials and Methods), a total of 105 samples from 41 dengue patients, 8 samples from healthy donors, and measurements for 10,075 unique probes (7,848 genes) from each of these samples were available for analysis.

**Table 1:** Characteristics of study participants with dengue.

	DF	DHF	DSS
	(n = 20)	(n = 12)	(n = 9)
Age (yrs) (range)	9.1 (0–14)	9.3 (2–14)	6.2 (0–12)
Gender: male/total (%)	9/20 (45)	5/12 (42)	3/9 (33)
First Sample Day	3.5 (2–5)	4 (3–5)	3.3 (2–5)
Dengue Type (%)			
DENV-1	5 (28)	0 (0)	1 (11)
DENV-2	13 (65)	12 (100)	8 (89)
Unknown	2 (10)	0	0
Immune Response (%)			
Primary	9 (45)	0 (0)	1 (11)
Secondary	11 (55)	11 (92)	8 (89)
Indeterminate	0	1 (8)	0
Thrombocytopenia (%)	0 (0)	12 (100)	9 (100)
Plasma Leakage (%)	5 (25)	12 (100)	9 (100)
Poor Capillary Refill (%)	5 (25)	3 (25)	9 (100)
Narrow Pulse Pressure (%)	7 (35)	1 (8)	9 (100)
Hypotension (%)	4 (20)	0 (0)	4 (44)
Cold Extremities (%)	10 (50)	7 (58)	7 (78)

Note: Data are median (range) unless otherwise indicated.

**Figure 1:**
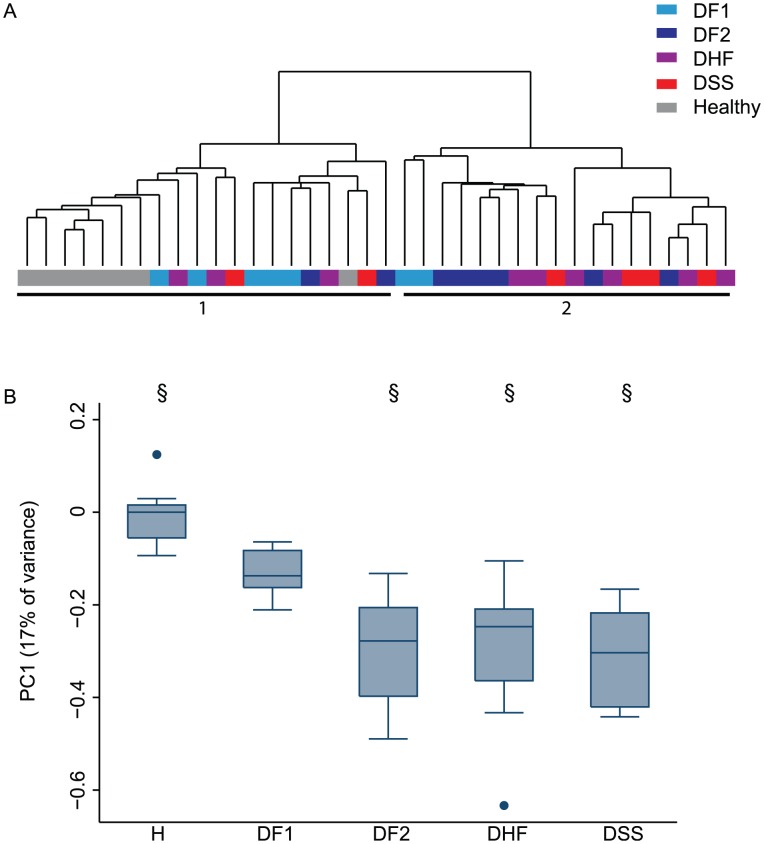
Gene expression in dengue patients on day 4 of fever. A) Hierarchical clustering of PBMC expression profiles from healthy individuals and dengue patients on fever day 4. Unsupervised clustering based on expression of 10,075 transcripts meeting quality control criteria (see Methods and Materials). Samples are colored by serologic status and WHO-defined clinical status. All patients with DHF or DSS had secondary DENV infection. Numbered horizontal bars refer to sample clusters described in the text. B) Values associated with principal component 1 from singular value decomposition of the data in (A). H = healthy control; DF1 = dengue fever, primary DENV infection; DF2 = dengue fever, secondary DENV infection; DHF = dengue hemorrhagic fever; DSS = dengue shock syndrome. §: differs from DF1 (p≤0.01, rank sum).

We used unsupervised hierarchical clustering to organize the data from the 30 samples collected from dengue patients on day 4 and the data from the 8 samples from healthy controls. One of the two major branches of the sample-based dendrogram included all healthy controls and 5 of 7 patients with DF1, while 16 of 18 samples in the other branch came from individuals with secondary DENV infection (DF2, DHF, and DSS) (Fig. 1A, clusters 1 and 2). Principal components analysis clarified the structure suggested by the clustering: despite marked differences in subsequent clinical outcome among the patients, the strongest source of variation in the data was the history of prior DENV infection. Healthy individuals were at one end of the continuum defined by the value of the first principal component (PC1), and patients with secondary infection were at the other; those with primary DENV infection had intermediate values of PC1 (Fig. 1B). The one DSS patient with primary infection, an infant believed to have maternal DENV-specific antibodies, had a PC1 score more similar to patients with classic primary infection than did any of the other DSS patients (Fig. S2).

**Figure 2:**
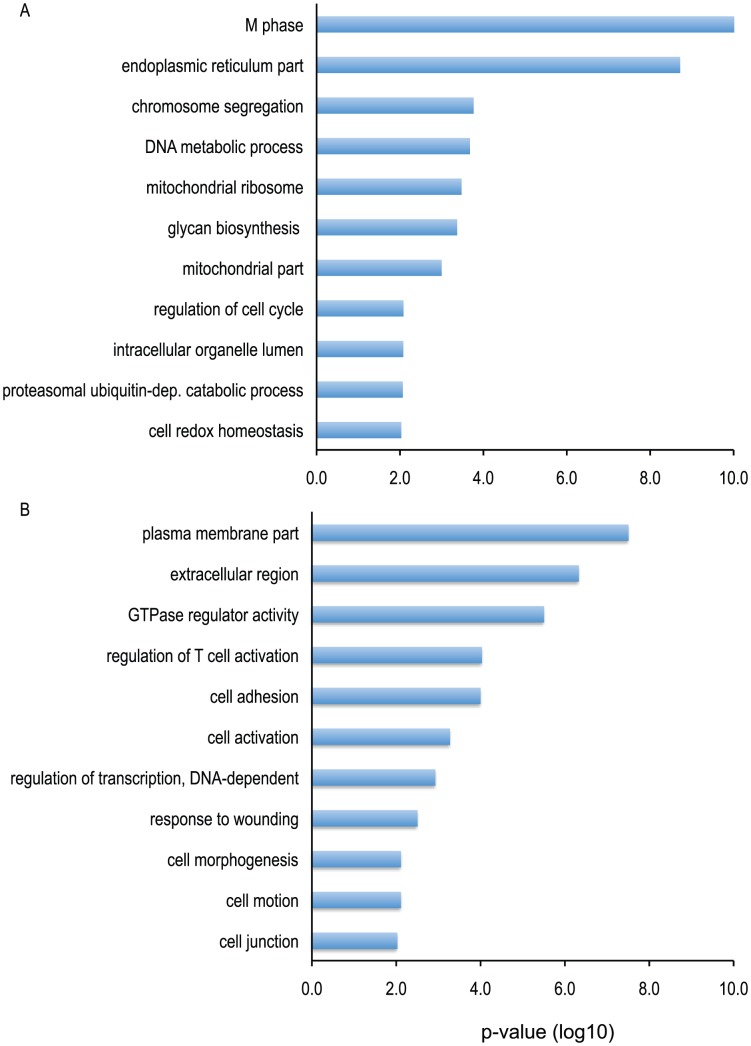
Gene ontologies associated with principal component 1 on fever day 4. Significant clusters of gene ontologies were identified using DAVID; only the most significant term in each GO cluster is shown, and only those with a p-value≤0.01. A) GO terms associated with a low PC1 score (DF2,DHF,DSS>DF1>H). B) GO terms associated with a high PC1 score (H>DF1>DF2,DHF,DSS).

To identify biological processes associated with PC1, we identified transcripts whose abundance was strongly correlated with this feature of the data, and then identified Gene Ontology (GO) terms linked to the associated genes (Fig. 2; see Methods). Terms associated with the mitotic cell cycle were strongly associated with PC1, and the corresponding gene transcripts were more abundant in patients with secondary DENV infection than with primary DENV infection (Fig. 2A). Other gene transcripts with greater abundance in secondary infection encode products associated with the endoplasmic reticulum, mitochondria, chromosome segregation, and metabolic homeostasis. Transcripts more abundant in healthy controls and in primary infection were annotated with GO terms related to cell activation and movement, including “plasma membrane part”, “extracellular region”, “regulation of T cell activation”, and “cell adhesion” (Fig. 2B).

### Temporal dynamics of the transcriptional response to DENV infection

The results of the unsupervised clustering indicated that patients with primary and secondary DENV infections had distinct transcriptional responses to DENV infection on day 4; we found the same distinction between these groups of patients when examining PC1 on fever days 3 and 5 (Fig. S3); therefore, we treated DF1 and DF2 as separate groups for the rest of our analyses. To characterize the evolution of the transcriptional response to DENV infection, we compared patterns of gene expression on each fever day in patients from each of the four categories described above (DF1, DF2, DHF, and DSS) to the patterns from eight healthy controls, using a false discovery rate of 1% as a threshold for significance and a re-sampling technique to control for differences in sample size [33].

**Figure 3:**
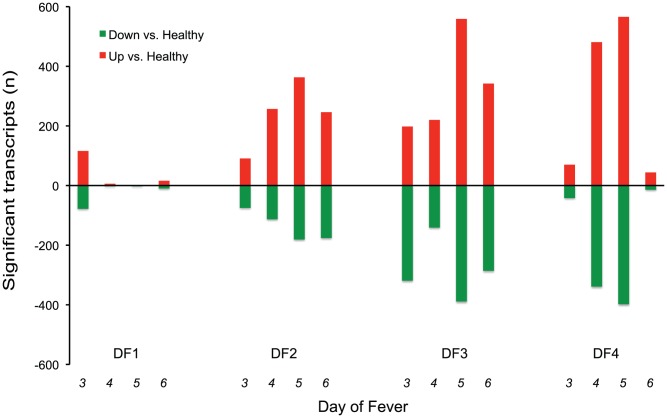
Transcripts with different abundance in each patient group compared to healthy individuals, by fever day. Significant differences in transcript abundance were identified using SAM [32] and a false discovery rate of 1%. Resampling was used to control for differences in sample size [33]. Red bars: transcripts more abundant in dengue patients than in healthy individuals; green bars: transcripts more abundant in healthy individuals.

**Figure 4:**
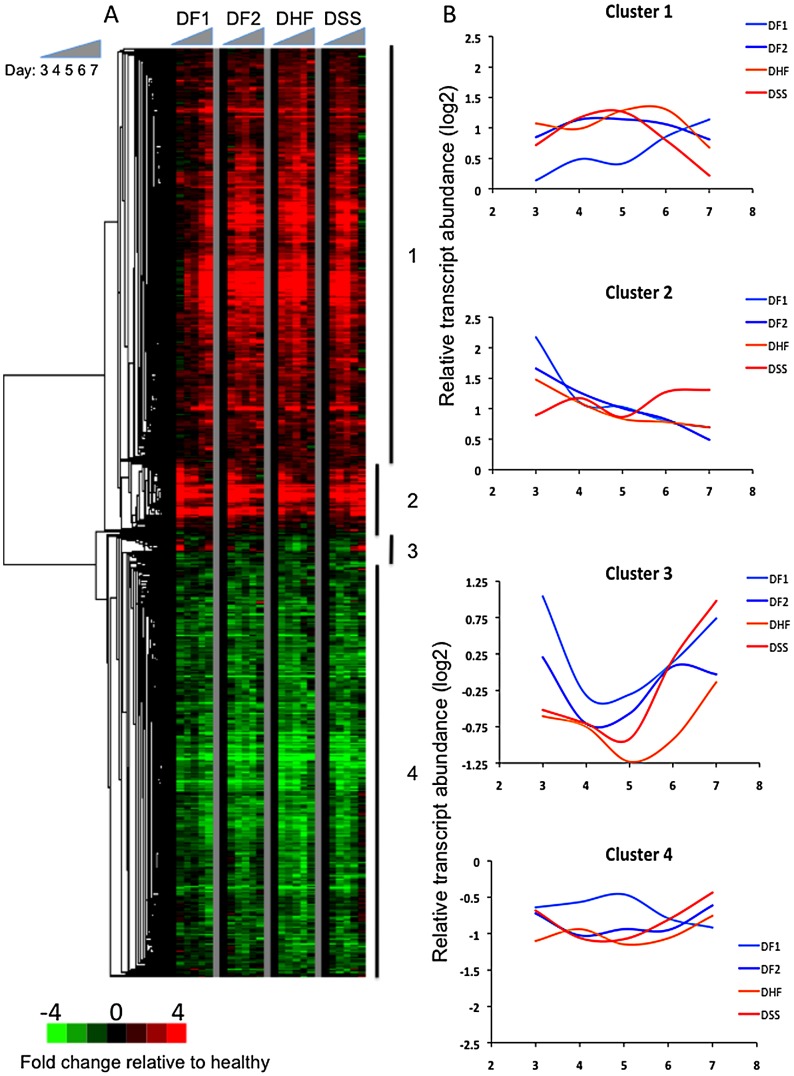
Differential gene expression between dengue patients and healthy controls, and among patient groups. A) Hierarchical clustering of 2,092 transcripts that differ in abundance in at least one patient group on fever days 3, 4, 5, or 6 when compared to healthy individuals. Median transcript abundance for each group on each of days 3 through day 7 (5 columns per group) is presented. Genes organized using hierarchical clustering, after normalizing to median value of healthy controls. Red indicates more abundant than controls; green indicates less abundant. Gray columns separate each group; the black column represents median value for healthy controls. Vertical lines and numbers 1–4 correspond to gene clusters discussed in the text. B) Median expression of each gene cluster, by day of illness and patient group.

The temporal pattern of transcript abundance differed dramatically among the patient groups (Fig. 3). Compared to healthy controls, 184 transcripts differed in abundance in patients with DF1 on fever day 3, but less than 10 transcripts were significantly different on days 4 and 5, and only 26 on day 6. In contrast, the number of differentially expressed transcripts peaked on day 5 in patients with secondary DENV infection. The day-to-day differences were particularly dramatic in patients with DSS; in these patients, 964 transcripts differed from healthy controls on day 5, but only 56 on day 6. In total, 2,092 transcripts demonstrated differences in abundance relative to healthy controls in at least one patient group on at least one day. To identify similarities and differences in gene expression in these patient groups, we calculated the median expression for each gene on each fever day in each group and used hierarchical clustering to organize these data. Four primary expression patterns emerged, each defined by an intra-cluster Pearson correlation coefficient of 0.5 or greater (Fig. 4A). Despite large differences in the number of differentially expressed transcripts among the patient groups, most genes were either consistently more or less abundant in all dengue patient groups relative to healthy controls across the time interval sampled. However, there were differences in the magnitude and timing of these expression patterns. Gene ontologies and pathways associated with each of these groups of genes were then examined.

Among the 2,092 transcripts that differed in abundance in at least one patient group on fever days 3, 4, 5, or 6 when compared to healthy individuals, the two largest clusters, 1 and 4, recapitulated the features identified on fever day 4 using principal components analysis, and distinguished DF1 from the other patient groups (Fig. 4B). Cluster 1 consisted of 959 transcripts with greater abundance in all patient groups compared to healthy controls, more so in those with secondary DENV infections. The abundance of these transcripts in patients with DF1 increased over time, but with different temporal patterns than in patients with secondary infection. Transcript levels were already elevated on day 3 in patients with secondary infection and began to decrease after day 5. Transcript abundance in DF1 patients on fever day 3 was lower than in the patients with secondary infections, but continued to increase at least through fever day 7. Cluster 1 was highly enriched for genes annotated with the GO terms “mitotic cell cycle” (p = 2.7E-16), “endoplasmic reticulum” (p = 2.8E-9), “chromosome condensation” (p = 5.5E-6) and “DNA synthesis and replication” (p = 2.9E-7), as well as mitochondrion (p = 5.4E-6). Genes in this cluster, including the immunoglobulin light chain gene (IGL), BLNK, and IRF4, are known to be highly enriched in lymphoblasts (p = 8.5E-110) [31].

The 941 transcripts in cluster 4 displayed an inverse pattern of expression compared to those in cluster 1, in that transcripts were less abundant in dengue patients than in healthy controls. This difference was again more pronounced in patients with secondary DENV infection. The nadir for expression of these genes in patients with secondary DENV infection occurred on day 5, after which transcript abundance increased, while levels in patients with DF1 continued to decrease after day 5. Genes in this cluster were enriched for GO terms associated with regulation of small GTPase-mediated signal transduction (p = 0.008), regulation of transcription (p = 0.02), and zinc finger proteins (SP keyword; p = 9E-4). T regulatory cells were the immune cell population most highly enriched for these transcripts (p = 4.9E-87) [31]. Together, the patterns of transcript abundance in clusters 1 and 4 suggest that a transient gene expression program suggestive of lymphocyte proliferation occurs in patients with secondary DENV infection that is evident by fever day 3 and proceeds more slowly, or starts later, in DF1 patients.

The two smaller gene clusters, 2 and 3, did not display the same dichotomous separation between DF1 and DF2 cases (Fig. 4B). The 151 transcripts in cluster 2 were elevated in abundance at all time points in all patients, compared to healthy controls, but exhibited divergent patterns associated with both dengue infection history and clinical outcome. On day 3, transcripts in cluster 2 were most abundant in DF1 patients and least abundant in DSS patients; by day 7, this pattern was reversed. Genes in this cluster encode proteins associated with antiviral defense and the RIG-I-like receptor (RLR) pathway for recognition and ubiquitination of viral nucleic acids, and proteins associated with induction of the interferon pathway (e.g., PLSCR1, ISG15, TBK1, and TRIM25). Twenty transcripts (15 genes) encoding histone proteins were among the most abundant transcripts in cluster 2. These genes encode all four of the canonical replication-dependent core histones (H2A, H2B, H3, and H4), as well as the replication-independent histones H1F0, H3.3B, and H2AFX. The presence of these histone transcripts led to enrichment of the GO term “nucleosome” (p = 6.9E-17); the Swiss-Prot keyword “antibiotic” was also significantly enriched by the presence of histone 2 transcripts (p = 0.018).

The 29 transcripts in cluster 3 exhibited the greatest range in abundance of the four gene sets, both in time and among the patient groups. There was a U-shaped pattern for median expression levels in all patient groups, with levels higher in patients with DF1 and DF2 and lower in those that developed more severe disease. Cluster 3 was associated with the GO term “membrane-bounded vesicle” (p = 0.06) and was highly enriched in platelet-related terms compared to other cell types (p<2.3E-28). In our dataset, levels of these transcripts were strongly correlated with platelet cell counts at the time the blood samples were collected for RNA analysis (p = 0.01; Fig. S4).

### Gene expression patterns associated with disease severity

**Figure 5:**
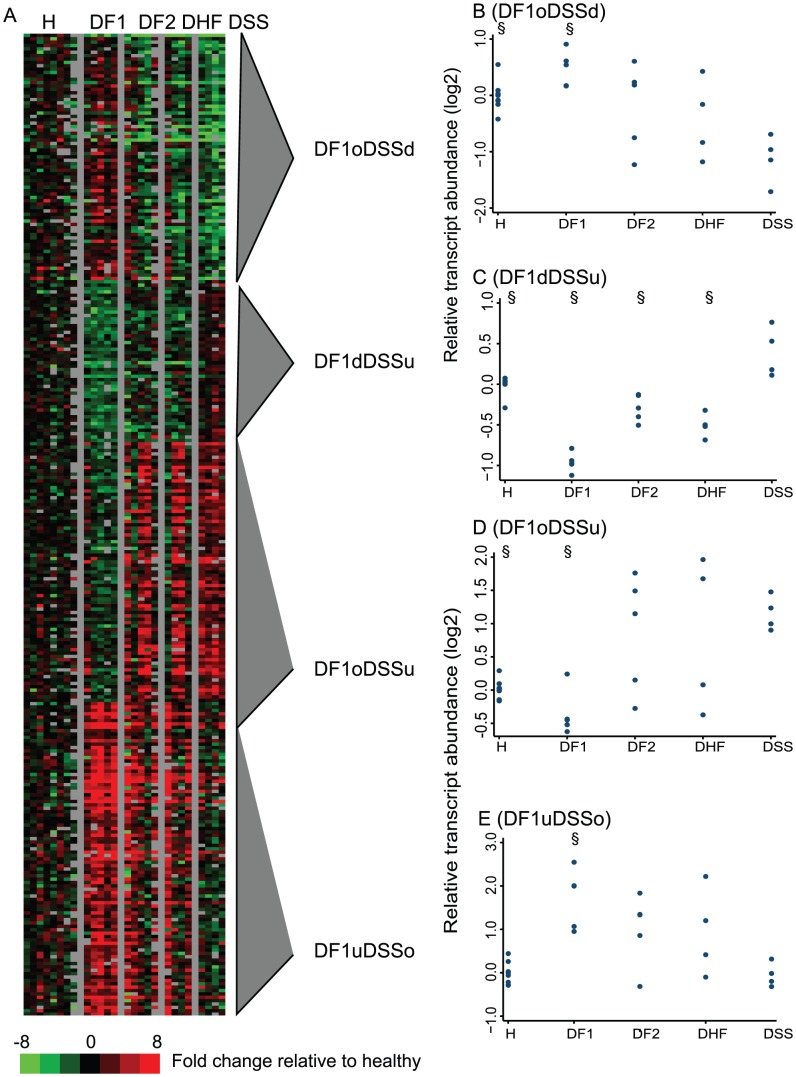
Transcripts whose abundance differed in patients with primary DF and secondary DSS on day 3. A) Abundance levels for 291 transcripts whose abundance differed between subjects with DF1 and subjects with DSS on fever day 3 (FDR<20%) were normalized to the median expression in healthy controls, and organized using k-means clustering. Gray triangles mark gene clusters that were annotated using expression relative to healthy individuals (u, significantly higher; o, not significantly different; d; significantly lower than healthy controls). B–E) Median expression of each gene cluster depicted in (A). Each data point represents a single patient. § indicates significant differences compared to patients with DSS (p<0.05, ranksum).

The temporal analysis was designed to identify differences between patient groups and healthy controls according to day of fever, rather than differences among the patient groups, but the results seen in clusters 2 and 3 (Fig. 4B) indicated that there might also be differences in gene expression linked to disease severity. We therefore examined the entire set of transcript abundance data for differences associated with the traditional WHO classification categories (DF, DHF, or DSS) [25] on each of fever days 3, 4 and 5. Using false discovery rates of less than 30%, there were no transcripts that distinguished between patients with DF2, DHF, and DSS, but there were 291 transcripts with a FDR<20% whose abundance differed between subjects with DF1 and subjects with DSS on fever day 3 (Fig. 5A and Table S1).

Four patterns defined the differences in gene expression among the DF1 and DSS patients on day 3. Two of these patterns were characterized by significant differences in transcript abundance between DSS patients, but not DF1 patients, and healthy controls (Figs. 5B and 5D, DF1oDSSu and DF1oDSSd). The other two were characterized by significant differences between DF1 and healthy controls (Figs. 5C and 5E, DF1uDSSo and DF1dDSSu). The first two patterns mirrored results from the temporal analysis and the unsupervised PCA, in that there were no significant differences in transcript abundance between patients with DF2, DHF, and DSS, and that the median expression in patients with secondary DENV infection differed significantly from both DF1 patients and healthy controls. The 77 transcripts with elevated abundance in secondary infections (Fig. 5B) have been previously reported to be more abundant in antigen-specific plasma B cells than in other B cells, as well as in activated CD8^+^ T cells compared to unactivated T cells; 55 of these transcripts were present in cluster 1 in the temporal analysis [34], [35]. The set of 73 transcripts that were less abundant in secondary infections (Fig. 5D) were enriched for transcripts associated with platelets, monocytes, and neutrophils (p = 8.9E-20, 2.3E-16, and 4.4E-5, respectively) and for genes annotated with GO terms associated with the innate immune response, including “response to wounding”, “inflammatory response”, and “regulation of cell communication”.

The third set of transcripts was more abundant in DF1 patients than in healthy controls, and similar in abundance between DSS patients and healthy controls (Fig. 5E). Levels in patients with DF2 and DHF were higher than in DSS patients, though this difference was only significant when DF2 and DHF patients were jointly compared to those with DSS (p<0.05). This set included 22 of the 151 transcripts identified in Cluster 2 in the temporal analysis, including histone 2A and 2B genes, and was highly enriched for genes encoding proteins involved in nucleosome organization (p = 6.18E-5). This set was also enriched for the Swiss-Prot keyword “antimicrobial” (p = 0.018), and for transcripts that are found in monocytes (p = 1.4E-14)

**Figure 6:**
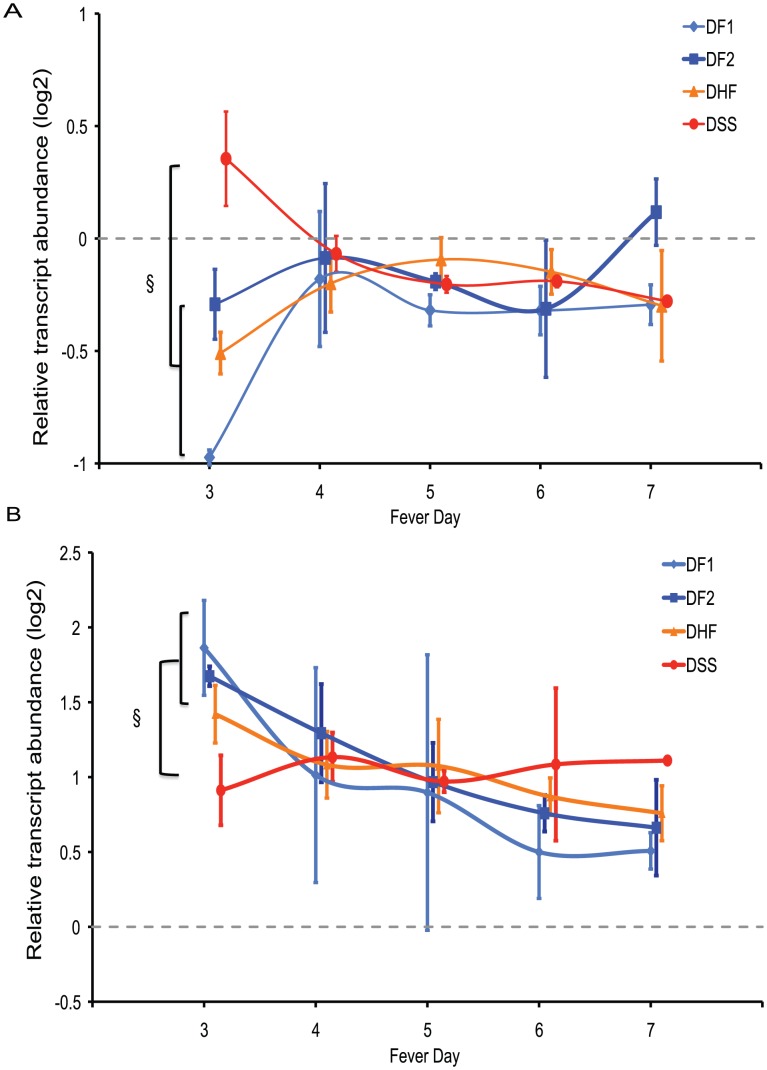
Temporal patterns of transcript abundance for genes associated with disease severity on fever day 3. A) Transcripts less abundant in DF1 patients and more abundant in DSS patients, compared to healthy controls (DF1dDSSu, Figure 5). B) PBMC transcripts induced by type-I interferons [37]. Symbols indicate median expression of all patients in the indicated clinical group sampled on that fever day. Light blue diamonds, DF1; dark blue squares, DF2; orange triangles, DHF; red circles, DSS. Error bars are defined by the median absolute deviation. Transcript abundance is calculated relative to the median abundance in healthy controls (dotted line). § indicates significant difference compared to DSS patients (p<0.05, ranksum).

The greatest difference in transcript abundance among the patient groups was observed with the fourth set of 48 transcripts (DF1dDSSu). In this set, transcript levels were lower in patients with DF1, DF2, and DHF than in healthy controls, while levels in patients with DSS were significantly higher than in healthy controls (Fig. 5B). Median expression of this group of genes not only differed between DF1 and DSS patients (p = 0.002), but also differed significantly when patients with secondary DF or secondary DHF were compared with those who developed DSS (p = 0.014 and p = 0.02, respectively). However, these differences in transcript abundance were not seen on days subsequent to fever day 3; median transcript levels in each patient group were not different from those in healthy controls on fever days 4 through 7 (Fig. 6A). This fourth set of genes was enriched for the GO term “mitochondrion” (p = 1.5E-50) and included a number of genes encoding structural components of the mitochondrion, including TOMM70A, c18orf22, WARS2, GLYCTK, GTPBP5, LYRM4, MTUS1, BCKDHB, ENOSF1, and SAMM50.

Multiple studies of dengue-related gene expression have reported lower levels of interferon-stimulated gene (ISG) expression in patients with more severe disease [8], [14], [36]. A previous study in patients hospitalized in Nicaragua, however, did not find an ISG expression signature associated with disease severity [13]. The temporal variation in gene expression observed in our study and the presence of ISGs in cluster 2 of the temporal analysis suggested that differences in ISG transcript abundance associated with disease severity might be evident on some days but not on others. To look for such a feature in our data, we took advantage of a previous study in which we defined ISG signatures by comparing gene expression patterns in PBMCs stimulated with type I IFNs and other cytokines [37]. Of the 150 genes identified in that study as responding similarly to three different type I interferons *in vitro*, 98 were represented in the dataset analyzed in the present study. Despite the different conditions *in vivo*, with shifting cell populations and multiple interacting stimuli, 57 of these genes, including canonical ISGs such as ISG15, ISG20, OAS2, ISI27, STAT1 and STAT2, had consistently elevated transcript levels in dengue patients compared to healthy controls (Fig. 6B). The median level of expression of these genes was also higher in each of the three other patient groups on fever day 3 compared to patients with DSS. However, as with the genes associated with mitochondrial function, these differences were absent on subsequent days (Fig. 6B).

## Discussion

We characterized the temporal evolution of the host transcriptional response to DENV infection in children hospitalized in Nicaragua. The dynamic and unpredictable clinical evolution of the disease led us to ask whether the host transcriptional response evolves in an analogous, dynamic fashion over a similar period of time, whether early features of the transcriptional response are correlated with subsequent clinical outcome, and whether temporal patterns were similar in primary and secondary DENV infection. We found differences in the timing and magnitude of gene expression that were related to both disease severity and a history of previous exposure to DENV.

Despite the range of clinical severity among the patients in this study, we found that history of past exposure to DENV, based on serology, was the greatest source of variation in gene expression among dengue patients. This feature of the data was maintained over multiple days of the acute illness, and led us to treat patients with primary and secondary DF as distinct molecular categories. The genes most strongly associated with this distinction were also highly enriched among a larger set whose transcript abundance patterns distinguished all dengue patients from healthy controls (p<0.001); thus, serological status was associated with differences in the timing and magnitude of a qualitatively conserved response to DENV infection. The gene most strongly associated with the first principal component of the gene expression matrix (Fig. 1) and the distinction between primary and secondary DENV infection was syndecan 1 (SDC1, or CD138), which is involved in the differentiation of B cells into antibody-producing plasmablasts and plasma cells at the level of both gene transcription and cell surface expression [34]. Other markers of plasma cell formation, including IRF4 and IGJ, were also strongly associated with the first principal component. Temporal expression of these genes lagged in patients with primary infection, and probably reflects the absence of memory B cells that lead to a rapid recall response in the patients with previous DENV infections. This transcript-based finding is consistent with recent flow cytometry-based studies that reported massive proliferation of plasmablasts within several days of the onset of symptoms in patients with dengue fever, particularly in those patients with secondary infection [38], [39].

Differences in transcript abundance associated with serological status suggest that the identification of transcripts associated with disease severity may be confounded by differences in the relative proportion of subjects with a previous history of DENV infection. Most previous studies of gene expression in dengue patients have been carried out in study populations in which all or nearly all patients had secondary DENV infections [8], [11], [12], [14], [15], [16]. Yet, the IgM and IgG serologic findings in a population of Brazilian dengue patients studied with DNA microarrays by Nascimento et al [36] suggest the presence of a mix of primary and secondary DENV infections. Twenty-four of the 40 genes whose transcript abundance in that study best differentiated DF and DHF were present in our filtered dataset and 6 were among the genes most strongly associated with the principal component that distinguished between primary and secondary DF (p<0.001, hypergeometric). These genes included the immunoglobulin lambda locus (IGL), MAGED1, TPD52, UBE2G1 and UBE2J1 – all of which we found more highly expressed in secondary DF than primary DF. In the Brazilian study, these same genes were more highly expressed in DF patients than in those with DHF; DF patients were more likely than DHF patients to be IgG-positive, suggesting that differences in seropositivity at the time of admission rather than disease severity may have been responsible for many of the observed differences in transcript abundance.

Despite the differences in transcript abundance profiles associated with serostatus, there were shared features in all four patient groups (Fig. 4). In order to determine whether these shared features were also found in the responses of humans to other acute viral infections, we first calculated the median expression levels across all samples collected between fever days 3 and 7 of genes with differential expression compared to healthy controls, and then searched for similarities between this set of transcript abundances and published blood-based expression patterns from relevant studies with healthy control subjects [7], [10], [15], [36], [40], [41], [42], [43], [44], [45], [46]. We found that the three most similar, previously-published expression patterns were from studies of DENV infection [15], [36], [44] (Fig. S5), even though these other studies were conducted in populations from different geographical regions using different types of samples from different time points in the course of illness, and different microarray platforms.

Gene transcripts induced by interferon were more abundant in all patients with acute DENV infection than in healthy controls (Fig. 4B, cluster 2 and Fig. 6A). This has been a consistent finding in studies that have compared PBMC or whole blood expression patterns in dengue patients with either healthy controls or convalescent timepoints from the same patients [8], [12], [15]. The strength of a corresponding association with disease severity has varied; it has been a prominent finding in some studies [8], [14], [16], [36] but not others [11], [12], [13]. These differences among previous studies in the reported prominence of an ISG signature associated with dengue clinical severity have been ascribed to differences in array platform and analytical approaches, as well as cell types used for analysis [11], [12]. Our identification of distinct temporal trends in interferon-stimulated gene expression in patients who developed DSS and in patients with less severe disease indicates that timing is also an important factor.

Surprisingly, genes encoding histone-associated proteins were co-expressed with the interferon-related transcripts (Fig. 4B, cluster 2). Transcripts of histone-encoding genes are typically not polyadenylated, and the mRNA amplification method we used requires a poly-A sequence for priming reverse transcription. However, a recent study described elevated levels of polyadenylated histone transcripts following G1 arrest [47], and type I interferons can induce G1 arrest in monocytes and other hematopoietic cells [48], [49].

Disease severity was also associated with differences on fever day 3 in the abundance of transcripts encoding mitochondrion-associated proteins. This set of transcripts was most abundant in patients who developed DSS, significantly less abundant in patients with DF2 and DHF, and least abundant in patients with DF1. This pattern suggests that biological processes involving mitochondrial function may be associated with the development of severe disease, and may contribute to the process by which secondary DENV infection increases the risk of DSS.

Establishing the mechanism represented by this mitochondrial signature associated with severe disease will require further study, but results of *in vitro* studies suggest that it may reflect the response to DENV-induced ER stress and the unfolded protein response (UPR) in infected cells [8], [50], [51]. Short-term adaptation to ER stress includes increased metabolic activity; continued stress can lead to autophagy or mitochondria-mediated apoptosis. Several studies have demonstrated that DENV infection induces specific forms of autophagy in hepatocytes that promote viral replication, perhaps by modulation of lipid metabolism [52]; however, in monocytes, pre-treatment of cells to induce ER stress and mitochondria-mediated autophagy resulted in lower levels of viral production [53]. Interestingly, 30 of the 44 genes in the DF1dDSSo cluster (Fig. 6) also demonstrated decreases in transcript abundance after treatment of neuroblastoma cells with atrazine, which promotes mitochondrial stress (p = 7.6E-6) [54]. ATF4, a transcription factor that positively regulates the pathway leading from phosphorylation of EIF2a to both autophagy and mitochondrial apoptosis was also expressed at higher levels on day 3 in patients who did not develop DSS.

A growing body of literature indicates that viral sensing systems and cellular responses to stress are integrated in mitochondria and that the latter help to regulate anti-viral responses, including the interferon system [55], [56]. It may be that mitochondrial apoptosis or autophagy represent a protective response to severe cellular stress and viral infection that results in diminished viral production, while continued or increased metabolic activity results in extended viral production and more severe disease. The decreased abundance of mitochondria-associated transcripts may define a conserved response to shock, as transcript levels were also lower in patients than in healthy controls in two studies of children with septic shock and one study of early-stage burn patients [57], [58], [59], and may provide new clues about mechanisms associated with shock.

Our choice of samples and analytic approach enabled us to identify temporal trends and differences between primary and secondary DENV infection, but also posed limitations. In order to avoid well-recognized artifacts that arise from amplification of RNA for array analysis on different days, we were careful to process all samples for direct comparison in parallel. This study design and technical issues restricted the sample size for analysis on any given fever day, and decreased our statistical power for identifying severity-associated patterns. In addition, we used PBMCs rather than whole blood. Previous studies of host gene expression during DENV infection have described a neutrophil-associated expression pattern associated with clinical outcome, and these cells were not present in our samples [11], [12], [13]. Samples with heterogeneous cellular composition, such as whole blood or PBMCs, may mask cell type-specific transcriptional features of interest; studies of specific cellular sub-populations, using either statistical or experimental approaches will almost certainly illuminate features of the host transcriptional response that predict disease severity.

Our finding of transcript abundance patterns on day 3, but not on later days, that were associated with subsequent disease severity raises the possibility that patterns predictive of disease severity may be apparent even earlier in the course of infection. Community-based studies at health centers and other settings where patients and even pre-symptomatic DENV-infected individuals are seen earlier in the disease process may provide an opportunity to identify such patterns. The data from this study highlight the importance of examining the changing temporal features of the host response more generally in the setting of acute infections.

## Supporting Information

Figure S1 **Distribution of PBMC RNA samples by day of fever.** DF1 = dengue fever, primary DENV infection; DF2 = dengue fever, secondary DENV infection; DHF = dengue hemorrhagic fever; DSS = dengue shock syndrome.
(DOCX)

Figure S2 **First principal component in the full gene expression data set for dengue patients on fever day 4 and healthy controls.** Principal components were derived from the dataset of 10,075 probes used for all analyses, including the data presented in Figure 1.
(PDF)

Figure S3 **First principal component in the data.** (A) Fever day 3. (B) Fever day 5. H = healthy control; DF1 = dengue fever, primary DENV infection; DF2 = dengue fever, secondary DENV infection; DHF = dengue hemorrhagic fever; DSS = dengue shock syndrome.
(PDF)

Figure S4 **Rank correlation of transcript abundance and platelet levels in dengue patients.** Median transcript abundance for each patient group on each of days 3 through day 7 (5 columns per group) is presented. Red indicates more abundant than controls; green indicates less abundant. Gray columns separate each group; the black column represents median value for healthy controls. Vertical lines and numbers 1–4 correspond to gene clusters identified in Fig. 2 discussed in the text. A moving average (window size = 11) of the Spearman rank correlation of platelet count and relative transcript abundance in all patient samples is presented on the right.
(PDF)

Figure S5 **Comparison of gene expression profiles in response to viral infection.** A dengue-response signature was constructed by calculating the median expression across all samples collected between fever day 3 and fever day 7 of genes with differential expression compared to healthy controls (Fig. 4). Studies including one or more comparisons of blood-based expression patterns in infected individuals and healthy controls were identified in Nextbio, and the associated list of differentially expressed genes was compared to the dengue-response signature from the current study. The length of the bar in each row indicates how similar each profile is to the profiles from the current study using the Nextbio measure of significance, which includes both the number of overlapping genes and the overall correlation of the patterns [31].
(DOCX)

Table S1 **Transcripts that are associated with differences between primary DF and secondary DSS on fever day 3 (FDR<20%).**
(XLS)
