# Supplementary material for: Temporal Dynamics of the Transcriptional Response to Dengue Virus Infection in Nicaraguan Children
Source: PLoS Negl Trop Dis. 2012 Dec 20;6(12):e1966. doi: 10.1371/journal.pntd.0001966 (PMC3527342; doi:10.1371/journal.pntd.0001966)
Supplement: Figure S1 — Distribution of PBMC RNA samples by day of fever. DF1 = dengue fever, primary DENV infection; DF2 = dengue fever, secondary DENV infection; DHF = dengue hemorrhagic fever; DSS = dengue shock syndrome. (DOCX) [file pntd.0001966.s001.docx]

Supporting Figure S1
