# Supplementary figures and images for: Temporal Dynamics of the Transcriptional Response to Dengue Virus Infection in Nicaraguan Children
Source: PLoS Negl Trop Dis. 2012 Dec 20;6(12):e1966. doi: 10.1371/journal.pntd.0001966 (PMC3527342; doi:10.1371/journal.pntd.0001966)

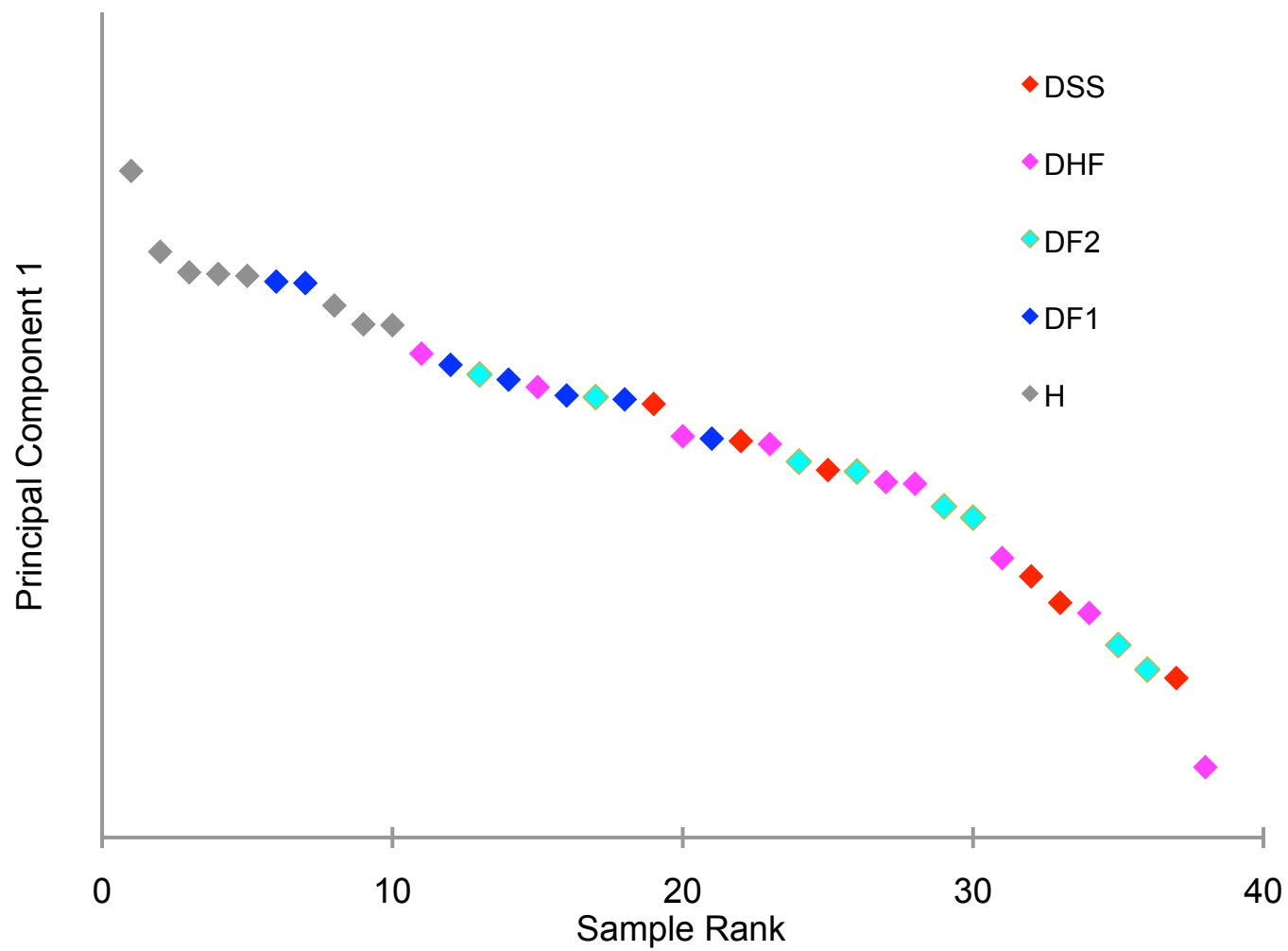

Supplement: Figure S2 — First principal component in the full gene expression data set for dengue patients on fever day 4 and healthy controls. Principal components were derived from the dataset of 10,075 probes used for all analyses, including the data presented in Figure 1. (PDF) [file pntd.0001966.s002.pdf]

A

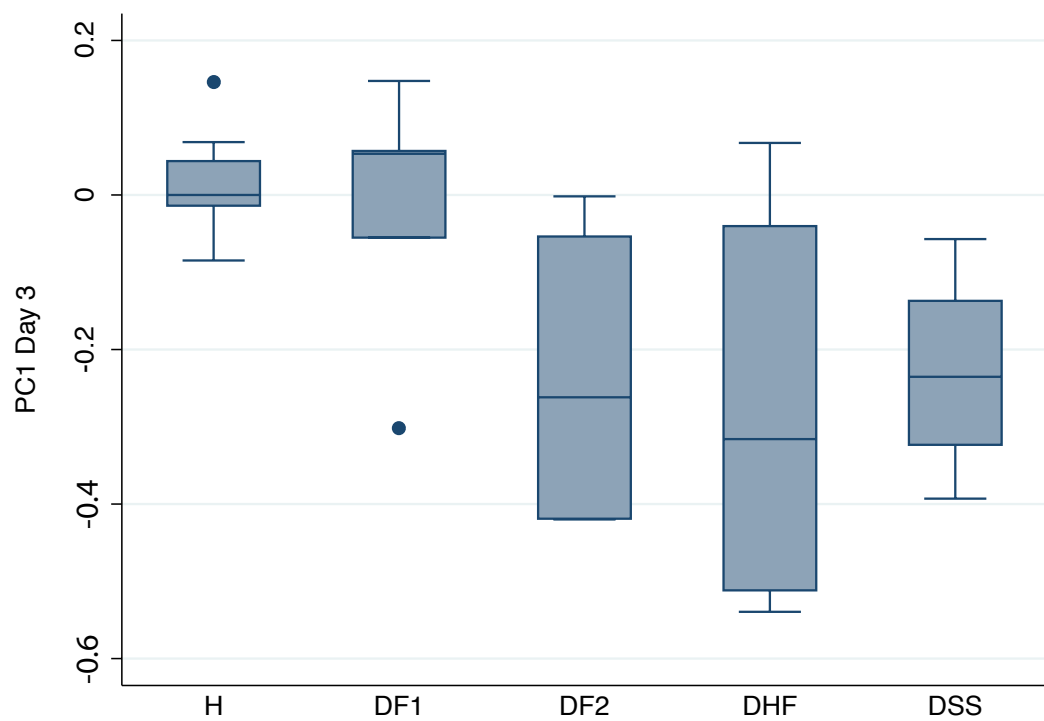

B

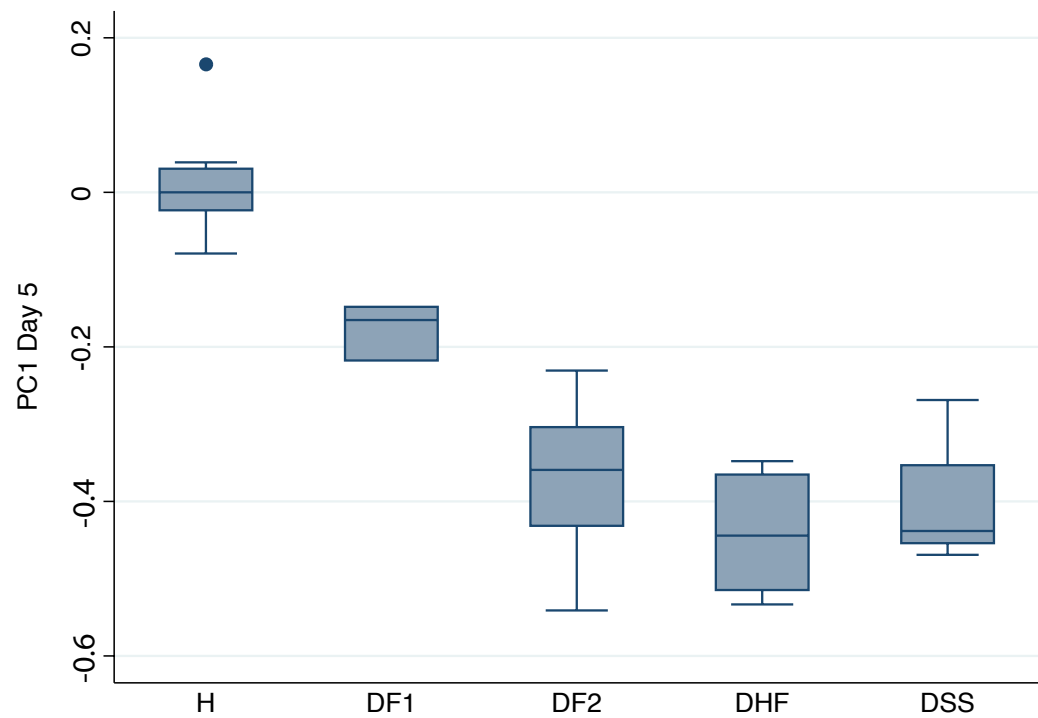

Supplement: Figure S3 — First principal component in the data. (A) Fever day 3. (B) Fever day 5. H = healthy control; DF1 = dengue fever, primary DENV infection; DF2 = dengue fever, secondary DENV infection; DHF = dengue hemorrhagic fever; DSS = dengue shock syndrome. (PDF) [file pntd.0001966.s003.pdf]

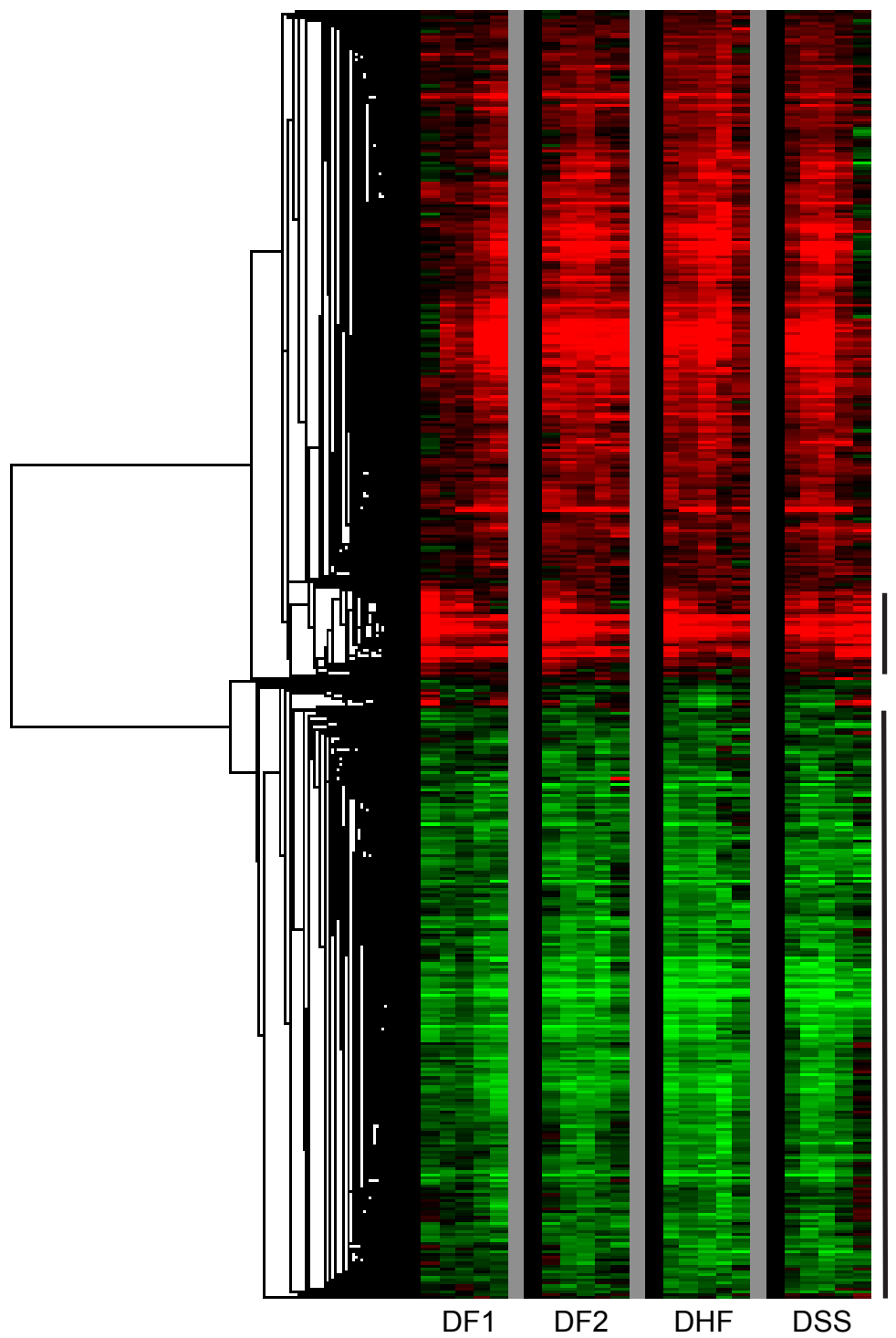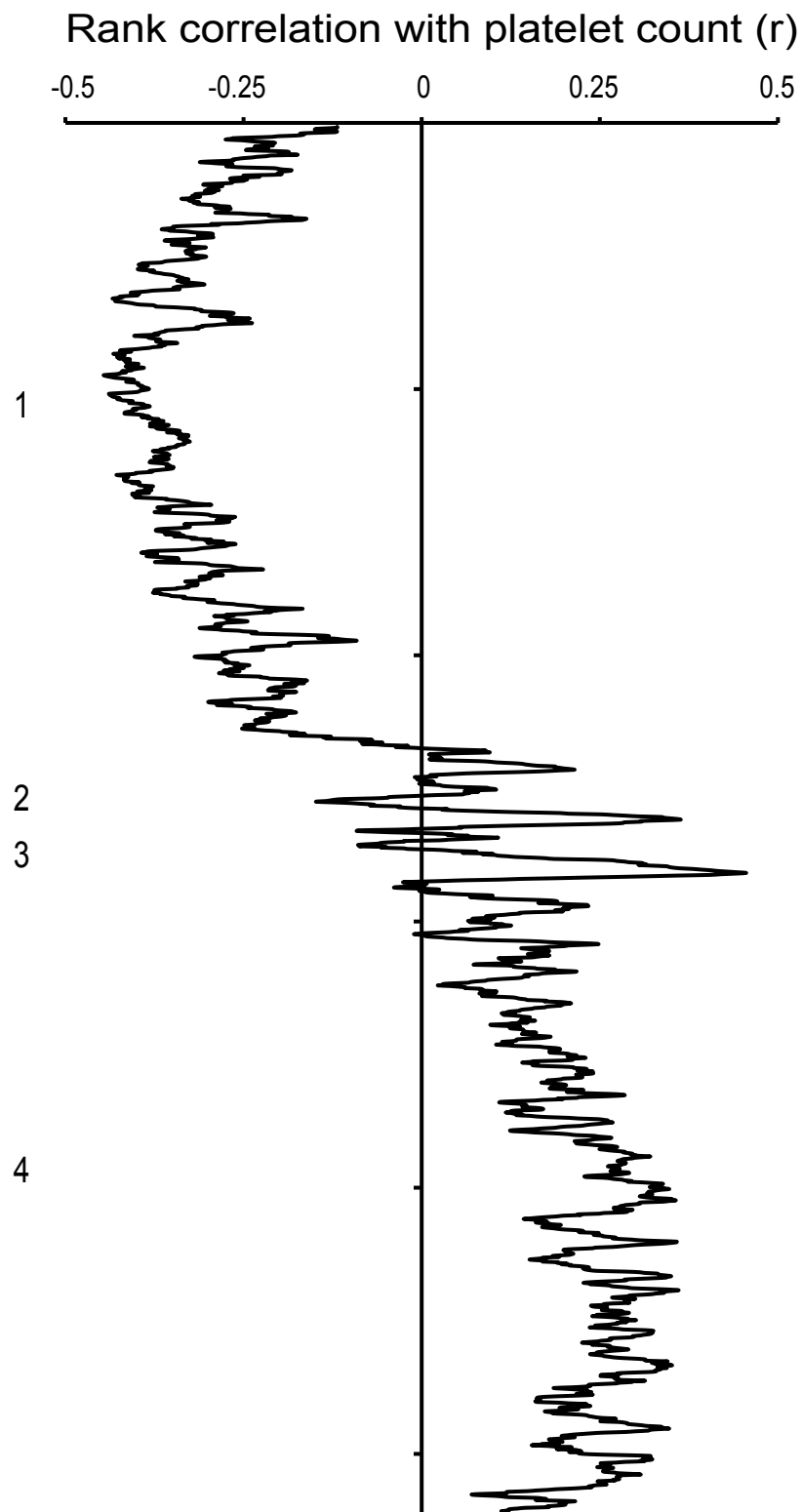

Supplement: Figure S4 — Rank correlation of transcript abundance and platelet levels in dengue patients. Median transcript abundance for each patient group on each of days 3 through day 7 (5 columns per group) is presented. Red indicates more abundant than controls; green indicates less abundant. Gray columns separate each group; the black column represents median value for healthy controls. Vertical lines and numbers 1–4 correspond to gene clusters identified in Fig. 2 discussed in the text. A moving average (window size = 11) of the Spearman rank correlation of platelet count and relative transcript abundance in all patient samples is presented on the right. (PDF) [file pntd.0001966.s004.pdf]
